# Metabolism-Related Genes SMOX and SUCLG2 as Immunological and Prognostic Biomarkers in Colorectal Cancer: A Pan-Cancer Analysis

**DOI:** 10.3390/cimb47060465

**Published:** 2025-06-17

**Authors:** Zuming Xiong, Yirong Lin, Yongjun Yang, Wenxin Li, Wei Huang, Sen Zhang

**Affiliations:** Department of Colorectal and Anal Surgery, The First Affiliated Hospital of Guangxi Medical University, Nanning 530021, China202020247@sr.gxmu.edu.cn (W.L.);

**Keywords:** prognosis, diagnosis, pan-cancer, colorectal cancer, tumor microenvironment, biomarkers

## Abstract

Expression patterns and underlying mechanisms of metabolism-related genes SMOX and SUCLG2 in pan-cancer remain unclear. We conducted a comprehensive pan-cancer analysis of SMOX and SUCLG2, to explore their potential roles and mechanisms of action. Comprehensive analysis of SMOX and SUCLG2 was performed through UCSC, TCGA, GEO, and other databases. We validated the expression levels, diagnostic value, and prognostic significance of SMOX and SUCLG2 in CRC using external databases and qPCR. Then, CCK-8 is used to detect proliferation of RKO and HCT116 after silencing or overexpressing of SUCLG2. The expression of SMOX was upregulated and that of SUCLG2 was downregulated in most cancers. Both SMOX and SUCLG2 exhibited significant correlations with cancer prognosis, tumor microenvironment, immune infiltration, stemness scores, tumor mutational burden, and microsatellite instability. The diagnostic and prognostic value of SMOX and SUCLG2 in CRC was confirmed through TCGA, GEO, and HPA, as well as qPCR. SUCLG2 overexpression inhibited the proliferation of RKO and HCT116, whereas SUCLG2 silence promoted their proliferation. Our data provide insights into the role of SMOX and SUCLG2 in pan-cancer, highlighting their association with prognosis, cancer immunity, and other cancer characteristics and also revealing their significance in cancer progression. SUCLG2 may inhibit the proliferation of CRC.

## 1. Introduction

The incidence of cancer is steadily increasing globally due to lifestyle changes and environmental degradation. Cancer has become the second leading cause of death worldwide, responsible for nearly one in six deaths [1]. Recent statistics reveal 19.3 million new cancer cases and approximately 10 million cancer-related deaths across the world, indicating that cancer poses a significant challenge to human health [2]. Accordingly, it has become imperative to explore more accurate early cancer detection technologies and improve cancer treatments to reduce the global burden of cancer. Precise diagnosis and personalized treatment approaches need to be prioritized, in addition to comprehensive research to elucidate the molecular mechanisms of oncogenes and antioncogenes [3].

Cells utilize various metabolic reactions to convert nutrients into small metabolite molecules in the cytoplasm [4]. Through further transformation, cells generate energy and essential substances that are vital for survival and cellular function [5]. When oxygen is abundant, cells primarily derive energy via mitochondrial oxidative phosphorylation, while in oxygen-deprived conditions, glycolysis becomes the main source of energy [6]. However, cancer cells tend to undergo glycolysis even in the presence of oxygen, a phenomenon known as the Warburg effect or aerobic glycolysis [7]. In addition, cancer cells suppress anticancer immune responses by competing for essential nutrients and reducing the metabolic adaptability of immune cells [8]. Abnormal metabolites and cancer-related metabolic intermediates also play an influential role in immune cell regulation [9]. Manipulating metabolism not only has the potential to enhance immune cell responses against cancer but also increase the immunogenicity of cancer cells, thereby expanding the range of cancers that can be effectively treated with immunotherapy [6].

The Kyoto Encyclopedia of Genes and Genomes database highlights the importance of spermine oxidase (SMOX) and succinate-CoA ligase GDP-forming subunit beta (SUCLG2. They are involved in diverse intracellular metabolic reactions and evidently related to cancer progression and prognosis. SMOX is a flavin adenine dinucleotide-containing enzyme and associated with the metabolism of polyamine; moreover, it is known to catalyze the oxidation of spermine to spermidine [10]. Polyamines play a key role in inflammation-induced carcinogenesis [11], but the role of SMOX seems to vary depending on the specific cancer. For instance, Tepper et al. found that SMOX expression was upregulated in lung, prostate, colon, stomach, and liver cancers and that it was associated with DNA damage, inflammation, and carcinogenic effects [12]. Furthermore, SMOX promotes gastric cancer by causing inflammation and DNA damage and activating beta-catenin signaling [13]. Fratini et al. indicated that SMOX expression was increased in neuroblastoma, regulating the proapoptotic genes BAX, BAK, and PUMA and supporting the antiapoptotic effect of AATF [14]. SUCLG2 encodes succinyl-CoA synthetase and participates in tricarboxylic acid cycle [15]. The tricarboxylic acid cycle and carbon metabolism play an essential role in cancer occurrence and development [16]. In prostate cancer cells, SUCLG2 enhances the succinic synthetase activity of mitochondrial nucleoside diphosphokinase and promotes leukemia suppressor receptor signaling to enhance prostate cancer aggressiveness [17]. In addition, SUCLG2 is associated with prognosis in clear cell renal cell carcinoma [18].

Pan-cancer analysis facilitates the identification of shared features and heterogeneity among various malignancies as it involves the simultaneous exploration of the expression and function of specific genes across different cancers. For example, a previous pan-cancer analysis found that HSF1 expression was significantly upregulated in various common cancers and that this upregulated expression was associated with poorer cancer prognosis [19]. Ferroptosis-related genes, such as SLC7A11, GPX4, and AIFM, have been used to evaluate immune cell infiltration in multiple cancer types [20]. EVA1B has shown prognostic and drug response associations with various cancers [21]. By employing a cross-cancer-type approach, pan-cancer analysis allows the extension of effective therapies from one cancer type to others with similar genomic characteristics, facilitating the identification of detection and treatment targets across different cancers [22].

SMOX and SUCLG2 play a vital role in cancerogenesis, particularly in cancer metabolism; however, their roles in pan-cancer remain unclear. Herein we analyzed SMOX and SUCLG2 expression trends in pan-cancer and explored the association of SMOX and SUCLG2 with cancer prognosis, immunity, cancer cell characteristics, and drug sensitivity. Moreover, we evaluated pathways related to SMOX and SUCLG2. To understand the clinical significance of SMOX and SUCLG2, we focused on colorectal cancer (CRC) for further analyses and predicted and constructed a potential competing endogenous RNA (ceRNA) network. Data were verified with external datasets and in vitro experiments.

## 2. Materials and Methods

### 2.1. Pan-Cancer Data and Expression and Correlation Analyses

Xena (https://xena.ucsc.edu/, accessed on 27 March 2020), a web-based visual integration and exploration platform created and maintained by the University of California Santa Cruz (UCSC), contains comprehensive data on the genomes of various organisms, such as humans, mice, and fruit flies [23]. We downloaded gene expression data, clinical information, immune profiles, and cancer stemness scores for 33 solid cancers from The Cancer Genome Atlas (TCGA) database through UCSC (comprising 11,057 samples derived from Adrenocortical carcinoma (ACC), Bladder Urothelial Carcinoma (BLCA), Breast invasive carcinoma (BRCA), Cervical squamous cell carcinoma, and endocervical adenocarcinoma (CESC), Cholangiocarcinoma (CHOL), Colon adenocarcinoma (COAD), Lymphoid Neoplasm Diffuse Large B cell Lymphoma (DLBC), Esophageal carcinoma (ESCA), Glioblastoma multiforme (GBM), Head and Neck squamous cell carcinoma (HNSC), Kidney Chromophobe (KICH), Kidney renal clear cell carcinoma (KIRC), Kidney renal papillary cell carcinoma (KIRP), Acute Myeloid Leukemia (LAML), Brain Lower Grade Glioma (LGG), Liver hepatocellular carcinoma (LIHC), Lung adenocarcinoma (LUAD), Lung squamous cell carcinoma (LUSC), Mesothelioma (MESO), Ovarian serous cyst adenocarcinoma (OV), Pancreatic adenocarcinoma (PAAD), Pheochromocytoma and Paraganglioma (PCPG), Prostate adenocarcinoma (PRAD), Rectum adenocarcinoma (READ), Sarcoma (SARC), Skin Cutaneous Melanoma (SKCM), Stomach adenocarcinoma (STAD), Testicular Germ Cell Tumors (TGCT), Thyroid carcinoma (THCA), Thymoma (THYM), Uterine Corpus Endometrial Carcinoma (UCEC), Uterine Carcinosarcoma (UCS), and Uveal Melanoma (UVM)). Gene expression data for cancer and normal samples were collated by Strawberry Perl, and information related to SMOX and SUCLG2 expression levels was acquired. A data matrix was constructed for subsequent analysis. All gene expression data were in FPKM format and converted to log2 values.

To investigate differences in gene expression between normal and cancer tissues, we employed the R package “limma”. Box charts and heatmaps were generated using the R packages “ggplot2” and “pheatmap”, respectively. Finally, the R package “corrplot” was used to assess the correlation between SMOX and SUCLG2 across different cancers.

### 2.2. Analyzing the Association of SMOX and SUCLG2 with Pan-Cancer Prognosis

For assessing the relationship between SMOX and SUCLG2 expression and patient prognosis, we integrated their expression levels with survival data. Based on optimized cutoff levels, patients were categorized into high- and low-expression groups. The Kaplan–Meier method, implemented with the R package “survival” was used to explore differences in prognosis between these groups, and survival curves were plotted. Furthermore, Cox regression analysis was performed to evaluate the correlation between SMOX and SUCLG2 expression levels and patient prognosis in various cancer types.

### 2.3. Analyzing the Association of SMOX and SUCLG2 with Pan-Cancer Cancer Immunity

Considering the close relationship between cancer metabolism and immunity, we analyzed the correlation between SMOX and SUCLG2 and immune-related indicators. The ESTIMATE algorithm was utilized to determine immune and stromal scores to predict the level of infiltrating immune and stromal cells in malignant cancer tissues. We calculated stromal, immune, and ESTIMATE scores; assessed tumor purity of each sample; and analyzed the correlation between SMOX and SUCLG2 and the four scores. In addition, CIBERSORT was employed to calculate the proportions of immune cell types and to analyze immune cell infiltration in cancers. We also evaluated the relationship between SMOX and SUCLG2 and immune cell infiltration across multiple cancer types.

Tumor mutational burden (TMB), microsatellite instability (MSI), and immunotype data were obtained from UCSC, and the correlation between each of them and SMOX and SUCLG2 was determined.

### 2.4. Analyzing the Association of SMOX and SUCLG2 with the Characteristics of Pan-Cancer Cells

Malta et al. proposed two algorithms for cancer stemness scores based on methylation and transcriptome data: the stemness score derived from methylation data reflects epigenetic characteristics (DNA stemness score, DNAss) and that from transcriptome data reflects gene expression (RNA stemness score, RNAss) [24]. We obtained two cancer stemness scores from UCSC and evaluated the relationship between SMOX and SUCLG2 expression and these scores.

Gene activity refers to the level of gene expression within cells, which can markedly affect the phenotype and fitness of an individual. To evaluate SMOX and SUCLG2 activities in all samples, we applied the single-sample gene set enrichment analysis algorithm. Furthermore, we compared gene activity between normal and cancer tissues in various cancers.

### 2.5. Drug Sensitivity Analysis

CellMiner™ (https://discover.nci.nih.gov/cellminer/home.do, accessed on 24 April 2020), a database and query tool created by the National Cancer Institute, facilitates the integration and study of molecular and pharmacological data for many cancerous cell lines. It contains information on 22,379 gene expression levels, 360 microRNAs, and 20,503 analyzed compounds [25]. SMOX and SUCLG2 expression in cell lines and drug sensitivity data were downloaded from CellMiner™. Subsequently, we investigated the relationship between drug sensitivity and SMOX and SUCLG2 expression levels.

### 2.6. Functional Enrichment Analysis

To conduct functional enrichment analysis, we used the “c2.cp.kegg.v7.4.gmt” pathway reference, which was derived from MSigDB [26] (http://www.gsea-msigdb.org/gsea/, accessed on 16 August 2021). This reference was utilized to investigate biological processes associated with SMOX and SUCLG2 across various cancers. Based on median SMOX and SUCLG2 expression levels, patients with cancer were classified into high- or low-expression groups. The R packages “GSVA” and “clusterProfiler” [27] were then used for gene pathway enrichment analysis [28,29,30], which facilitated the evaluation of differences in biological processes between the high- and low-expression groups.

### 2.7. Association with CRC, External Validation, and ceRNA Network Construction

To further investigate the close association of SMOX and SUCLG2 with CRC, we performed a comprehensive analysis examining their correlation with immunotypes, clinical stages, and ESTIMATE scores specific to CRC.

CRC datasets were obtained from the Gene Expression Omnibus (http://www.ncbi.nlm.nih.gov/geo/, accessed on 27 March 2023) database [31] to validate the multidimensional expression of SMOX and SUCLG2. Gene sequencing results from blood samples of healthy individuals and patients with CRC (GSE10715 and GSE174302), as well as tissues from normal and CRC cases (GSE39582 and GSE44076), were utilized to verify the trends in SMOX and SUCLG2 expression levels. The diagnostic ability of SMOX and SUCLG2 in CRC was evaluated using receiver operating curve (ROC) analysis and area under the curve (AUC) calculations.

In addition, we validated CRC data from TCGA. Using the Xiantao online tool (https://www.xiantaozi.com/, accessed on 28 March 2023), we compared SMOX and SUCLG2 expression in normal and CRC tissues, evaluating their diagnostic and prognostic (optimal grouping method) predictive values for CRC.

The Human Protein Atlas (HPA, https://www.proteinatlas.org, accessed on 27 April 2023) database, which contains information pertaining to RNA and protein expression levels in a wide range of tissues and organs [32], was used to verify differences between SMOX and SUCLG2 protein expression levels across tissues.

To explore the regulatory mechanisms of SMOX and SUCLG2 in CRC, we predicted and constructed a ceRNA network. Potential target miRNAs of SMOX and SUCLG2 were predicted by ENCORI [33], miRDB [34], mirTarBase [35], miRWalk [36], and TargetScan [37]. We subjected miRNAs predicted in at least three databases and that were differentially expressed in CRC to further analyses. Subsequently, we predicted potential target lncRNAs for the aforementioned miRNAs using ENCORI and intersected them with differentially expressed lncRNAs to obtain target lncRNAs. Finally, the ceRNA network was constructed.

### 2.8. qPCR

For qPCR, CRC and adjacent normal colorectal tissues were collected from patients at the First Affiliated Hospital of Guangxi Medical University from March 2022 to May 2023. Overall, 56 pairs of tissues were collected from patients who underwent surgery at our hospital, and written informed consent was obtained from all patients prior to tissue collection. This study was approved by the Ethics Committee of the First Affiliated Hospital of Guangxi Medical University.

The collected samples were stored at −80 °C until needed. Total RNA was extracted using TRIzol (B511321; Sangon Biotechnology, Shanghai, China). cDNA was synthesized with SweScript RT II First Strand cDNA Synthesis Kit (G3333, Servicebio, Wuhan, China). Gene expression was measured using 2× Universal Blue SYBR Green qPCR Master Mix (G3326, Servicebio). Relative gene expression was normalized to GAPDH and calculated using the comparative threshold cycle (2^−△△Ct^) method.

The following primer sequences were used: GAPDH-forward: CAGGAGGCATTGCTGATGAT and GAPDH-reverse: GAAGGCTGGGGCTCATTT; SMOX forward: CGGATGACCCTCTCAGTCG and SMOX reverse: GCGTGTCCAAGTTTCACACT; SUCLG2 forward: CAAAAGACCCTAATGTTGTGGGA and SUCLG2 reverse: TTCAGCAACCATCACCTTGTT.

Furthermore, we collected information pertaining to the clinical pathological stage of the corresponding patients to analyze the correlation between SMOX and SUCLG2 and clinical pathological stages.

### 2.9. Cell Culture, Transfection and CCK-8 of SUCLG2

The human CRC cell lines RKO, and HCT116 were purchased from Haixing Biosciences (Suzhou, Jiangsu, China). RKO, and HCT116 cells were cultured in RPMI-1640 medium (Gibco, New York, NY, USA). All culture media were supplemented with 10% fetal bovine serum (FBS) (VivaCell, Shanghai, China) and 1% (penicillin/streptomycin (P/S; Gibco). Cells were cultured in standard cell culture incubators at 37 °C with 5% CO_2_.

For transient silence or overexpression of CRC cells, small interfering RNA (siRNA, Hanbio, Shanghai, China) or Plasmid DNA (Miaoling Biotechnology, Wuhan, China) was transfected into CRC cells using 0.15 µL (per 96-well plate) or 4 µL (per 6-well plate) Lipofectamine 3000 (Invitrogen, Carlsbad MFG, USA). Additional P3000 needs to be added during Plasmid DNA transfection (0.1 µL per 96-well plat, 2 µL per 6-well plate). The cells for testing transfection efficiency through qPCR were harvested 48 h after transfection. Sequences of SUCLG2 siRNA were as follows: sense (5′-3′): GGUACAAUCUAGCGACAAATT; antisense (5′-3′): UUUGUCGCUAGAUUGUACCTT. Plasmid DNA (p-SUCLG2) is NM_001177599.2 Transcript Variant of SUCLG2.

Cell viability was assessed using the Cell Counting Kit-8 (CCK-8; UElandy,Suzhou, Jiangsu, China) according to the manufacturer’s protocol. Cells were seeded into 96-well plates at a density of 1000 cells/well. Cells that were silenced or overexpressed were cultured for 96 hours and 72 h, respectively. The optical density (OD) value was measured at 450 nm using a microplate reader after 2 h of incubating.

### 2.10. Statistical Analysis

All statistical analyses were performed using R v4.2.2. Spearman or Pearson correlation analysis was employed to evaluate the relationship between variables. Differences between groups were analyzed using the Wilcoxon test. *p* < 0.05 indicated statistical significance (“*” represents *p* < 0.05; “**” represents *p* < 0.01; and “***” represents *p* < 0.001).

## 3. Results

### 3.1. Differential Expression Analysis and Correlation Between SMOX and SUCLG2 Across Different Cancers

**Figure 1:**
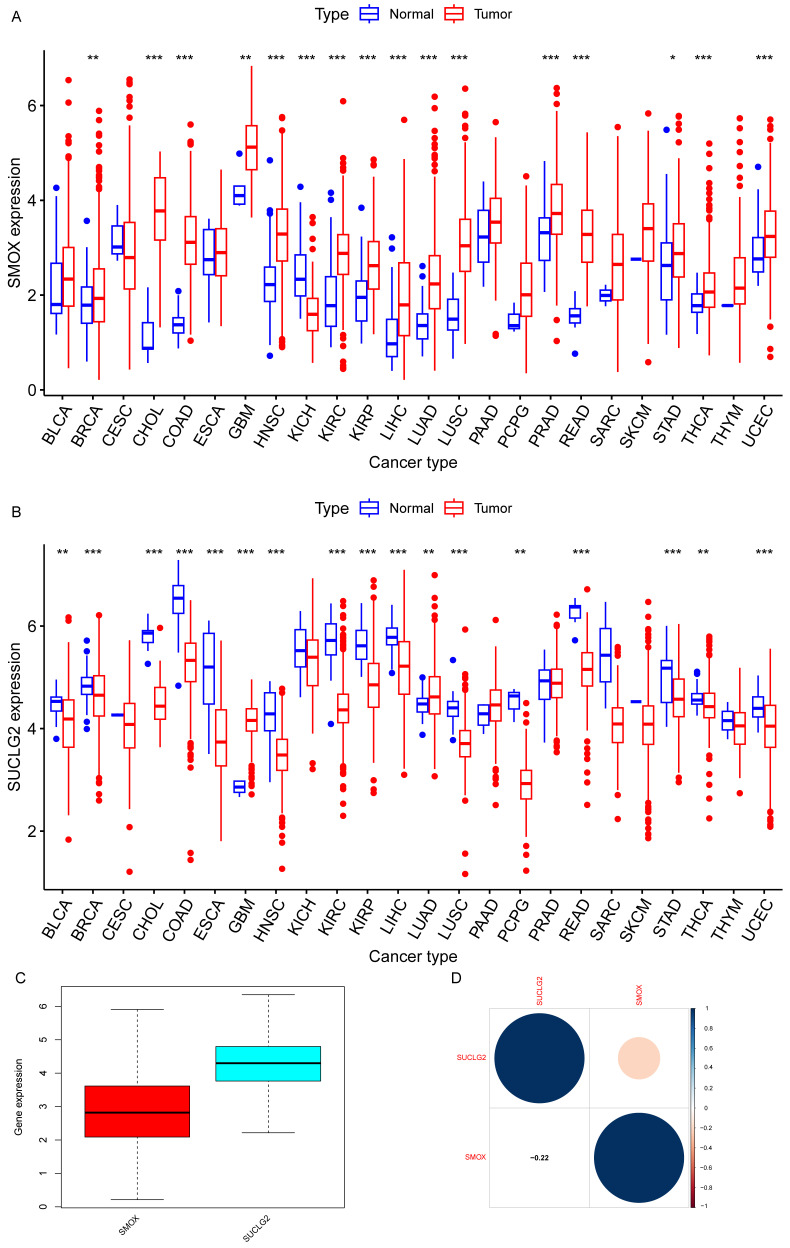
(**A**) SMOX and (**B**) SUCLG2 expression in pan-cancer. (**C**) The expression levels of SMOX and SUCLG2 in pan-cancer. (**D**) Association between SMOX and SUCLG2 expression. “*” represents *p* < 0.05; “**” represents *p* < 0.01; and “***” represents *p* < 0.001.

For pan-cancer analysis, we selected cancers with at least one normal sample available for examination. Our findings revealed differential expression of SMOX in BRCA, CHOL, COAD, GBM, HNSC, KICH, KIRC, KIRP, LIHC, LUAD, LUSC, PRAD, READ, STAD, THCA, and UCEC (*p* < 0.05). The expression of SMOX was upregulated in most cancers, except KICH (Figure 1A). Similarly, SUCLG2 displayed differential expression in BLCA, BRCA, CHOL, COAD, ESCA, GBM, HNSC, KIRC, KIRP, LIHC, LUAD, LUSC, PCPG, READ, STAD, THCA, and UCEC (*p* < 0.05). Its expression was downregulated in most cancers, except GBM and LUAD (Figure 1B). In this manner, a negative correlation was observed between SMOX and SUCLG2 expression in pan-cancer, consistent with gene expression patterns (Figure 1C,D).

### 3.2. Prognostic Significance of SMOX and SUCLG2 in Pan-Cancer

**Figure 2:**
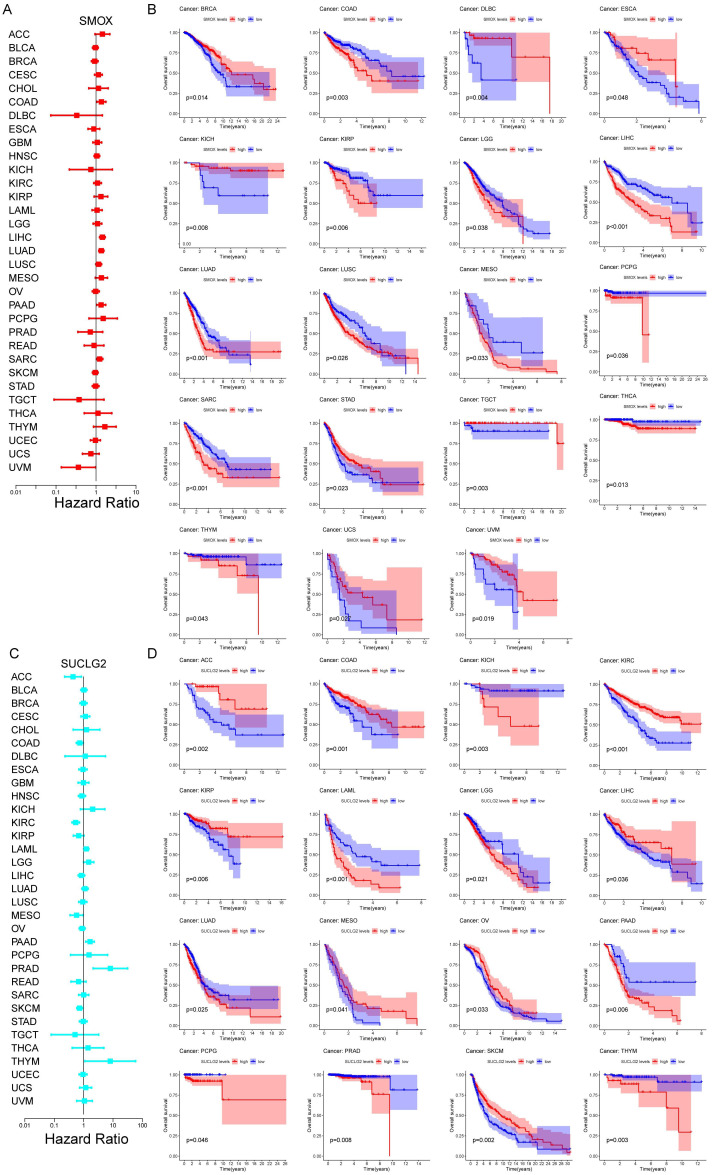
Pan-cancer survival analysis. (**A**) Cox regression analysis results based on SMOX expression in pan-cancer. (**B**) The Kaplan–Meier survival curves of pan-cancers between the high and low SMOX expression groups; (**C**) Cox regression analysis results based on SUCLG2 expression in pan-cancer. (**D**) The Kaplan–Meier survival curves of pan-cancers between the high and low SUCLG2 expression groups.

In pan-cancer survival analyses, the results of Cox regression analysis revealed key findings. SMOX emerged as a prognostic risk factor for COAD, LIHC, LUAD, PAAD, and SARC and prognostic protective factor for UVM (Figure 2A). Notably, in BRCA, COAD, DLBC, ESCA, KICH, KIRP, LGG, LIHC, LUAD, LUSC, MESO, PCPG, SARC, STAD, TGCT, THCA, THYM, UCS, and UVM, there were significant differences in prognosis between the high and low SMOX expression groups, with the majority of patients with high SMOX expression experiencing worse prognosis (Figure 2B). On the other hand, SUCLG2 emerged as a prognostic protective factor for ACC, COAD, KIRC, MESO, and SKCM and prognostic risk factor for LAML, PAAD, and THYM (Figure 2C). In ACC, COAD, KICH, KIRC, KIRP, LAML, LGG, LIHC, LUAD, MESO, OV, PAAD, PCPG, PRAD, SKCM, and THYM, prognosis markedly varied between the high and low SUCLG2 expression groups, with the majority of patients with low SUCLG2 expression showing worse prognosis (Figure 2D).

### 3.3. Relationship Between SMOX and SUCLG and Pan-Cancer Cancer Immunity

There are mainly six cancer immunophenotypes, which are C1 to C6. SMOX and SUCLG2 were found to be strongly associated with pan-cancer immunophenotypes (*p* < 0.001). SMOX expression was the highest in C5 and lowest in C3, while SUCLG2 expression was the highest in C3 and lowest in C5 (Figure S1A).

**Figure 3:**
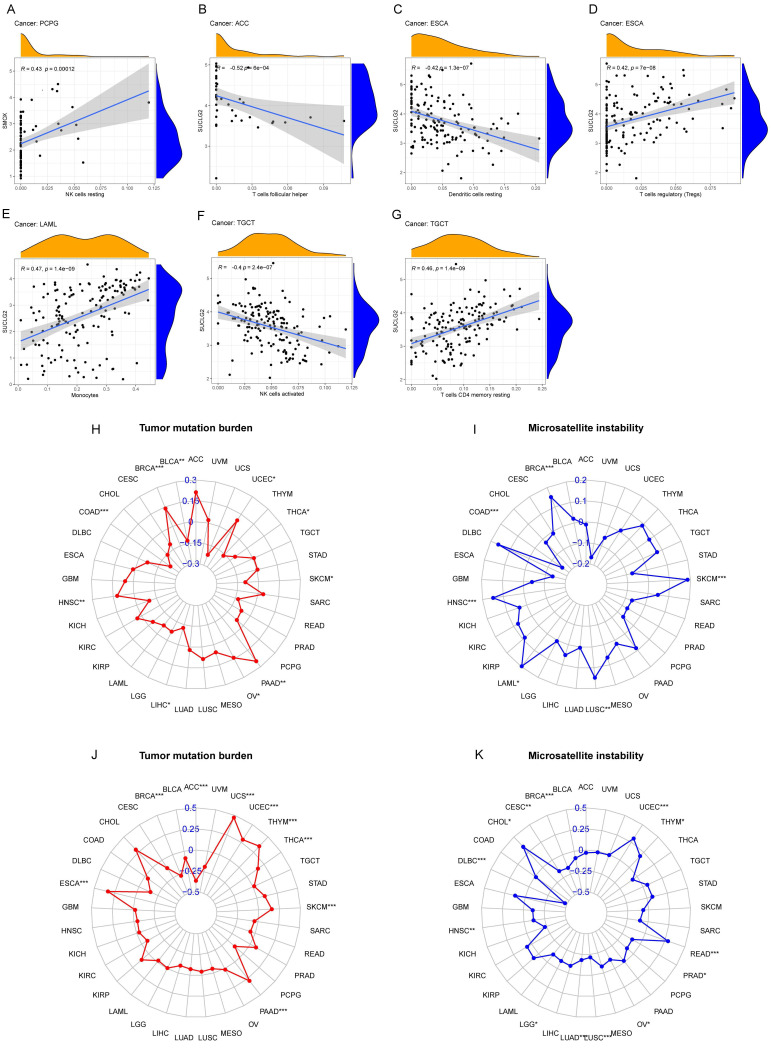
(**A**–**G**) SMOX and SUCLG2 correlation analysis with immune cells. Correlation of SMOX (**H**,**I**) and SUCLG2 (**J**,**K**) with TMB and MSI. “*” represents *p* < 0.05; “**” represents *p* < 0.01; and “***” represents *p* < 0.001.

In the analysis of the tumor microenvironment (TME), SMOX and SUCLG2 showed close associations with ESTIMATE, immune, and stromal scores, and with tumor purity in COAD, READ, and several other cancers (*p* < 0.05). For example, ESTIMATE scores of BLCA, BRCA, CESC, HNSC, KICH, LGG, LIHC, LUSC, PAAD, PCPG, PRAD, SKCM, STAD, and TGCT were related to SMOX (*p* < 0.05), with most of them showing negative correlations. SUCLG2 exhibited the strongest correlation with ESTIMATE scores of BLCA, CHOL, COAD, GBM, HNSC, KIRC, KIRP, LAML, LGG, LIHC, LUAD, LUSC, OV, PAAD, PCPG, READ, TGCT, THCA, and UCEC (*p* < 0.05), with most of them displaying negative correlations (Figure S1B–E). Immune scores of BLCA, BRCA, CESC, HNSC, KICH, KIRC, LGG, LIHC, LUSC, OV, PAAD, PCPG, PRAD, SKCM, STAD, TGCT, and THCA were related to SMOX (*p* < 0.05). Immune scores of BRCA, CHOL, COAD, GBM, HNSC, KIRC, KIRP, LAML, LUAD, LUSC, OV, PCPG, PRAD, READ, THCA, THYM, UCEC, and UCS were related to SUCLG2 (*p* < 0.05). Stromal scores of BLCA, KICH, KIRC, LGG, LIHC, LUSC, MESO, PAAD, PCPG, PRAD, READ, STAD, and THYM were related to SMOX (*p* < 0.05). Stromal scores of BLCA, BRCA, CESC, COAD, GBM, HNSC, KIRC, KIRP, LAML, LIHC, LUAD, LUSC, PAAD, PCPG, READ, SARC, TGCT, THCA, THYM, UCEC, and UVM were related to SUCLG2 (*p* < 0.05). Tumor purity of BLCA, BRCA, CESC, HNSC, KICH, LGG, LIHC, LUSC, PAAD, PCPG, PRAD, SKCM, STAD, and TGCT were related to SMOX (*p* < 0.05). Tumor purity of BLCA, CHOL, COAD, GBM, HNSC, KIRC, KIRP, LAML, LIHC, LUAD, LUSC, OV, PAAD, PCPG, READ, TGCT, THCA, and UCEC were related to SUCLG2 (*p* < 0.05). In addition, immune cell infiltration analyses indicated that SMOX was positively correlated with resting natural killer (NK) cells; SUCLG2 was negatively correlated with T follicular helper cells, resting dendritic cells, and activated NK cells and positively correlated with regulatory T cells, resting memory CD4 T cells, and monocytes (Figure 3A–G).

In pan-cancer tissues, SMOX was associated with TMB in COAD, BRCA, PAAD, HNSC, BLCA, LIHC, OV, THCA, UCEC, and SKCM and with MSI in BRCA, SKCM, COAD, HNSC, LUSC, and LAML. SUCLG2 was associated with TMB in ACC, BRCA, ESCA, PAAD, SKCM, THCA, THYM, UCEC, and UCS and with MSI in BRCA, CESC, CHOL, DLBC, HNSC, LGG, LUAD, LUSC, OV, PRAD, READ, THYM, and UCEC (Figure 3H–K).

### 3.4. Relationship Between SMOX and SUCLG2 and Pan-Cancer Cancer Characteristics

**Figure 4:**
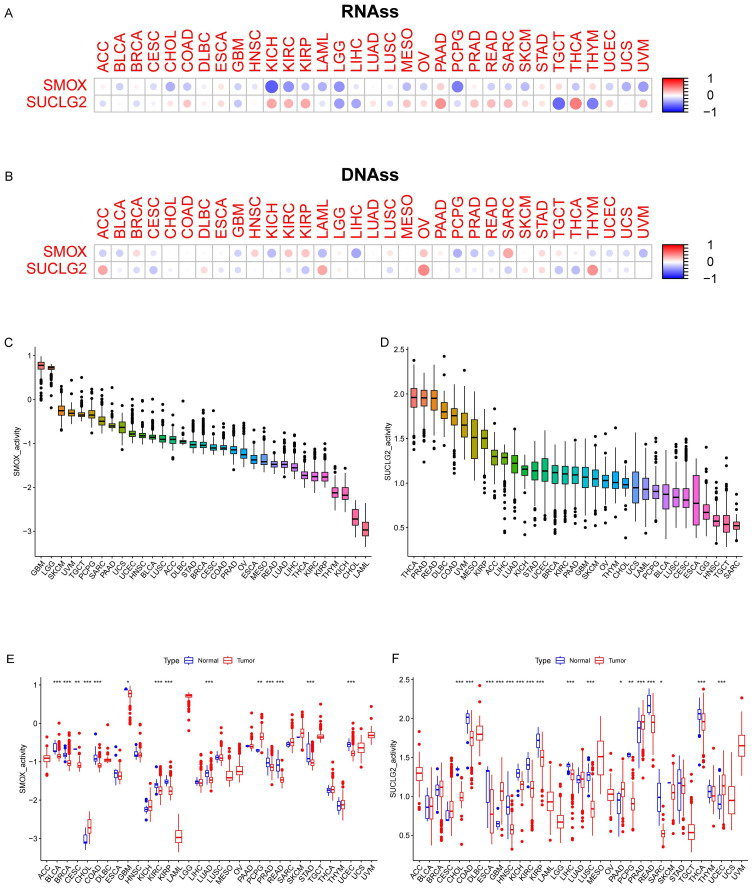
(**A**) Correlation analysis results of SMOX and SUCLG2 with RNAss and (**B**) DNAss in pan-cancer. (**C**,**D**) SMOX and SUCLG2 activity in pan-cancer. (**E**,**F**) Differences in SMOX and SUCLG2 activity in pan-cancer. “*” represents *p* < 0.05; “**” represents *p* < 0.01; and “***” represents *p* < 0.001.

Correlation analyses indicated that SMOX was positively correlated with RNAss in ACC, BRCA, ESCA, LUAD, PRAD, STAD, TGCT, and THYM (*p* < 0.05) and negatively correlated with RNAss in CHOL, DLBC, LIHC, and LUSC (*p* < 0.05). Furthermore, SUCLG2 was positively correlated with RNAss in COAD, KICH, KIRC, KIRP, LUAD, OV, PAAD, PRAD, READ, SARC, SKCM, STAD, THCA, UCEC, and UVM (*p* < 0.05) and negatively correlated with RNAss in BRCA, GBM, LGG, LIHC, LUSC, PCPG, TGCT, and THYM (*p* < 0.05) (Figure 4A). With regard to DNAss, correlation analyses produced similar results for SMOX and SUCLG2 (Figure 4B).

In addition, SMOX activity was the highest in GBM and lowest in LAML, differing from normal tissues in cancers such as COAD, BLCA, and READ. On the other hand, SUCLG2 activity was the highest in THCA and lowest in SARC, differing from normal tissues in cancers such as CHOL, COAD, and READ, with most of them showing a downward trend (Figure 4C–F).

### 3.5. Association of SMOX and SUCLG2 with Drug Sensitivity

**Figure 5:**
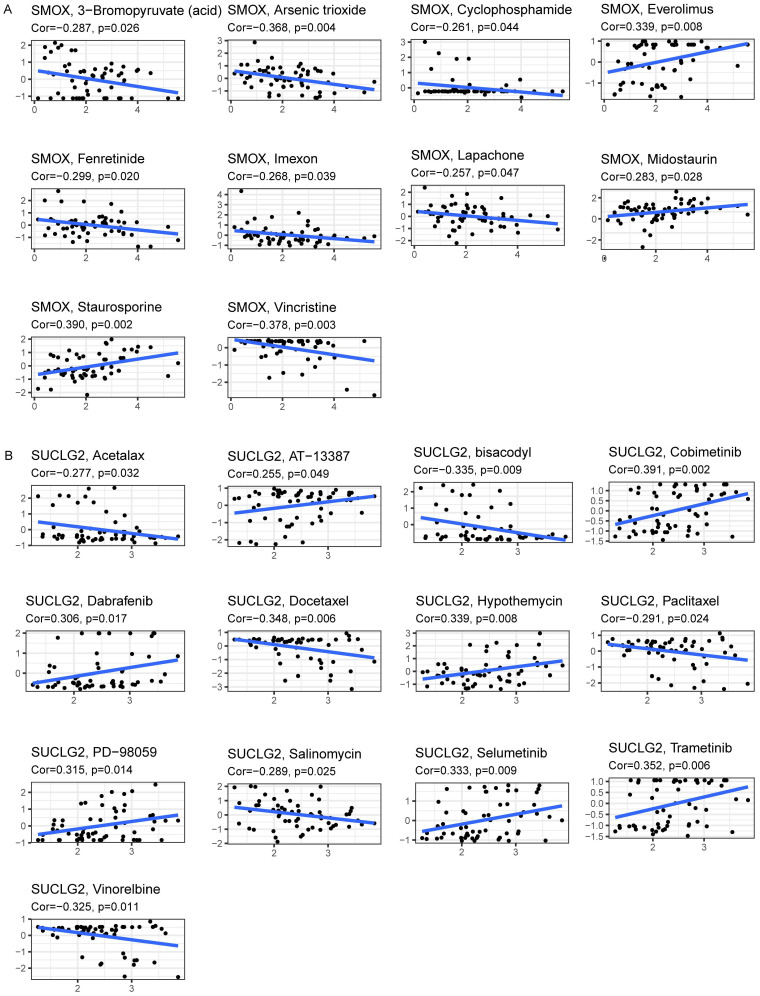
Drug sensitivity analysis with significant correlation baesed on CellMiner. (**A**) Data for SMOX and (**B**) SUCLG2.

SMOX was closely related to the sensitivity of various drugs, including 3-bromopyruvate (acid), arsenic trioxide, cyclophosphamide, everolimus, fenretinide, imexon, lapachone, midostaurin, staurosporine, and vincristine. SMOX expression was positively correlated with everolimus, midostaurin and staurosporine and negatively correlated with the other drugs (Figure 5A). On the other hand, SUCLG2 expression displayed negative correlations with drug sensitivity of acetalax, bisacodyl (active ingredient of viraplex), docetaxel, paclitaxel, salinomycin, and vinorelbine and was positively correlated with AT-13387, cobimetinib (isomer 1), dabrafenib, hypothemycin, PD-98059, selumetinib, and trametinib (Figure 5B).

### 3.6. Functional Enrichment Analysis

**Figure 6:**
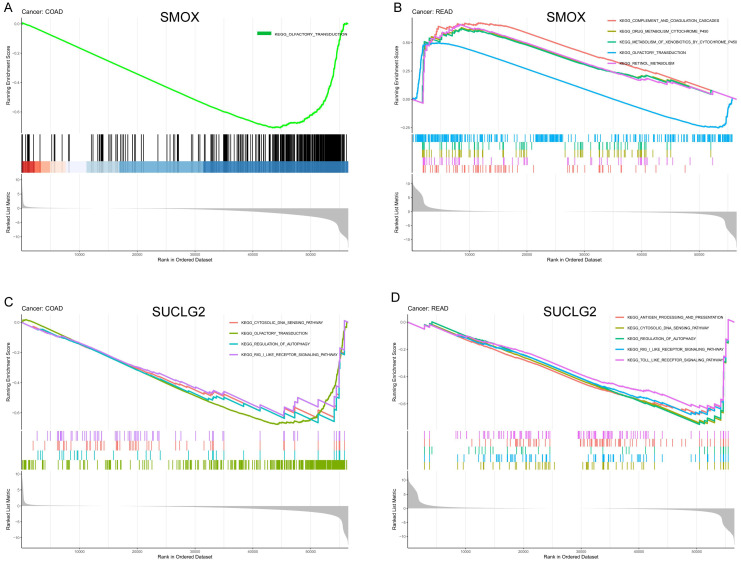
(**A**) Enrichment analysis results for SMOX in COAD and (**B**) READ. (**C**) Enrichment analysis results for SUCLG2 in COAD and (**D**) READ.

According to gene set enrichment analysis, SMOX and SUCLG2 were associated with various pathways related to olfactory conduction, metabolism, and immunity, suggestive of their involvement in diverse carcinogenic pathways. In COAD, pathway analysis results indicated that lower SMOX expression was associated with olfactory transduction (Figure 6A). Moreover, in READ, high SMOX expression was associated with pathways such as drug metabolism cytochrome P450, complement and coagulation cascades, and retinol metabolism (Figure 6B).

In COAD, low expression of SUCLG2 was associated with pathways such as cytosolic DNA-sensing pathway and regulation of autophagy (Figure 6C), while in READ, low expression of SUCLG2 was associated with pathways such as the toll-like receptor signaling pathway, antigen processing and presentation, and drug metabolism (Figure 6D).

### 3.7. Association of SMOX and SUCLG2 with CRC

**Figure 7:**
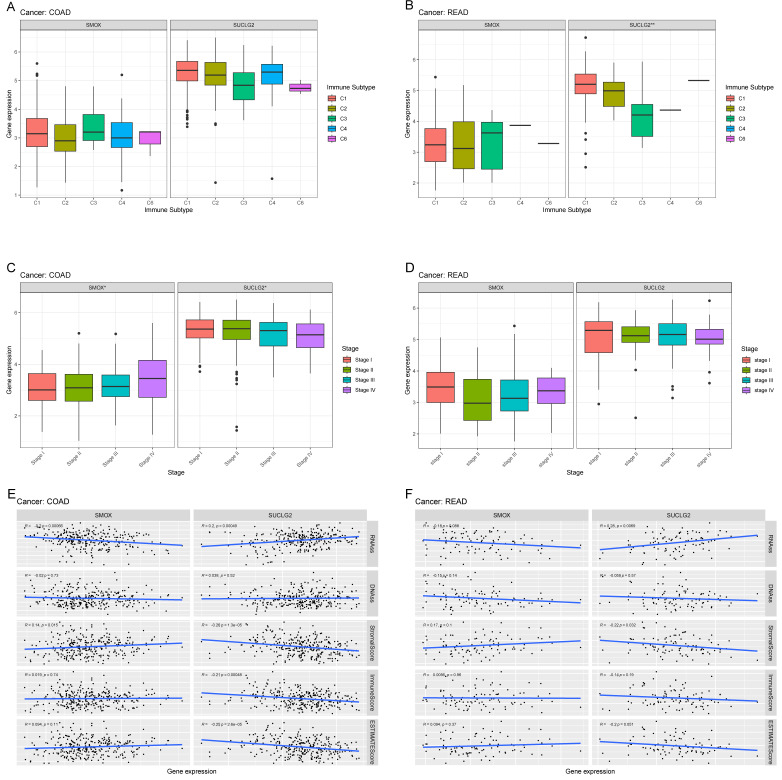
Correlation results for SMOX and SUCLG2 with the (**A**,**B**) immunophenotypes, (**C**,**D**) stages, and (**E**,**F**) TME scores of COAD and READ.

We found that SMOX was not associated with immunophenotypes of COAD or READ, while SUCLG2 was associated with immunophenotypes of READ, showing high expression in C1, and relatively low expression in C3. There was no significant correlation between SUCLG2 and COAD immunophenotypes (Figure 7A,B).

In COAD, SMOX and SUCLG2 were correlated with clinicopathological stages. As the clinicopathological stage advanced, the expression of SMOX gradually increased while that of SUCLG2 decreased. However, no significant correlations were found between SMOX and SUCLG2 and READ clinicopathological stages (Figure 7C,D).

In COAD, SMOX was negatively correlated with RNAss (R = −0.2, *p* < 0.001) and positively correlated with stromal score (R = 0.14, *p* < 0.05). In contrast, SUCLG2 was positively correlated with RNAss (R = 0.2, *p* < 0.001) and negatively correlated with stromal (R = −0.26, *p* < 0.001), immune (R = −0.21, *p* < 0.001), and ESTIMATE (R = −0.25, *p* < 0.001) scores (Figure 7E). In READ, SMOX did not show significant correlations with RNAss, DNAss, or any scores in TME. On the other hand, SUCLG2 was positively correlated with RNAss (R = 0.28, *p* < 0.01) and negatively correlated with stromal score (R = −0.22, *p* < 0.05) (Figure 7F).

### 3.8. External Validation of SMOX and SUCLG2 in CRC with Gene Expression Omnibus and TCGA Datasets

**Figure 8:**
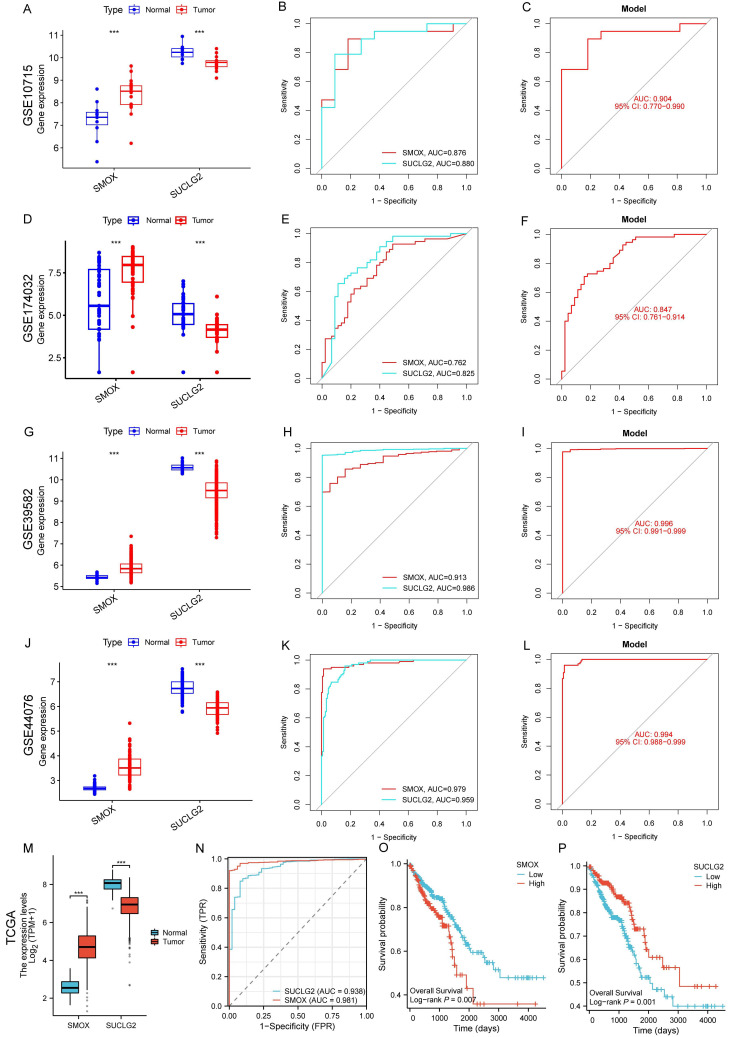
Expression differences (**A**,**D**,**G**,**J**), independent (**B**,**E**,**H**,**K**) or combined model (**C**,**F**,**I**,**L**) diagnostic ability of SMOX and SUCLG2 in GSE10715, GSE174032, GSE39582, and GSE44076. For TCGA dataset, results related to expression differences (**M**), diagnostic ability (**N**), and Kaplan–Meier survival curves (**O**,**P**) in CRC are shown. “***” represents *p* < 0.001.

In blood (GSE10715 and GSE174032) and tissue (GSE39582 and GSE44076) samples, relative to the normal groups, the expression of SMOX increased while that of SUCLG2 decreased in the cancer groups. When evaluating the diagnostic ability of CRC, the AUC of ROC for SMOX in GSE10715 and GSE174032 was >0.76, the AUC for SUCLG2 was >0.82, and the AUC for the diagnostic model combining SMOX and SUCLG2 was >0.84. The AUCs for SMOX and SUCLG2 in GSE39582 and GSE44076 were >0.90 and those for the diagnostic models combining SMOX and SUCLG2 were >0.99. These results indicated that SMOX and SUCLG2 have excellent diagnostic capabilities for CRC (Figure 8A–L).

Similar outcomes were observed in TCGA dataset, where SMOX expression increased while SUCLG2 expression decreased in cancer tissues (Figure 8M). Both SMOX and SUCLG2 showed good diagnostic ability for CRC (Figure 8N). Survival analysis revealed that patients with high SMOX expression, and those with low SUCLG2 expression exhibited worse prognosis (Figure 8O,P).

### 3.9. CeRNA Network Establishment

To establish a ceRNA network, we predicted 21 SMOX-related and 104 SUCLG2-related miRNAs using six databases. Among them, 27 were differentially expressed in CRC. We identified 25 potential target lncRNAs with differential expression in CRC and then constructed a ceRNA regulatory network consisting of 25 lncRNAs, 20 miRNAs, and 2 mRNAs (Figure S2A).

### 3.10. Validation by HPA and qPCR

**Figure 9:**
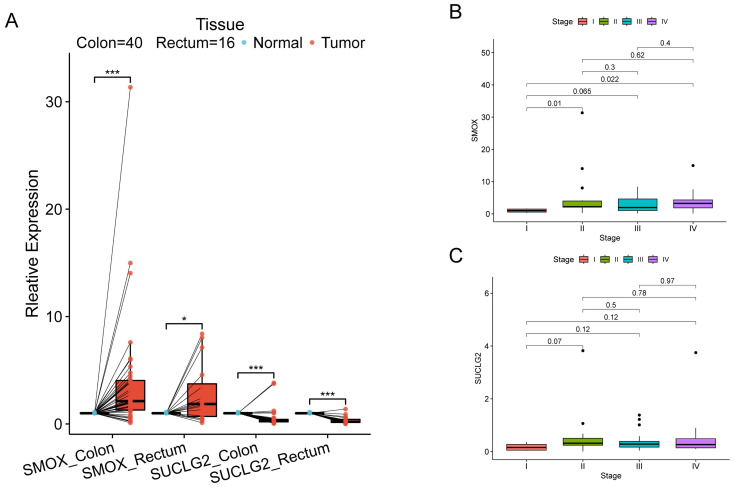
(**A**) qPCR results for SOMX and SUCLG2 in CRC and adjacent normal colorectal tissues. (**B**,**C**) Correlation analysis between SMOX and SUCLG2 and pathological stage. “*” represents *p* < 0.05; and “***” represents *p* < 0.001.

HPA verified high expression of SMOX and low expression of SUCLG2 in CRC tissues (Figure S2B–E). SMOX and SUCLG2 expression levels in 56 pairs of CRC and adjacent normal colorectal tissues were measured by qPCR, which indicated that relative to normal tissues, SMOX was increasingly expressed and SUCLG2 was decreasingly expressed in cancer tissues (*p* < 0.05) (Figure 9A).

Correlation analyses revealed that SMOX expression was significantly different only between stage I and stages II and IV, while there was no significant difference in SMOX and SUCLG2 expression among the other stages (Figure 9B,C).

### 3.11. SUCLG2 Inhibited the Proliferation of CRC Cells

**Figure 10:**
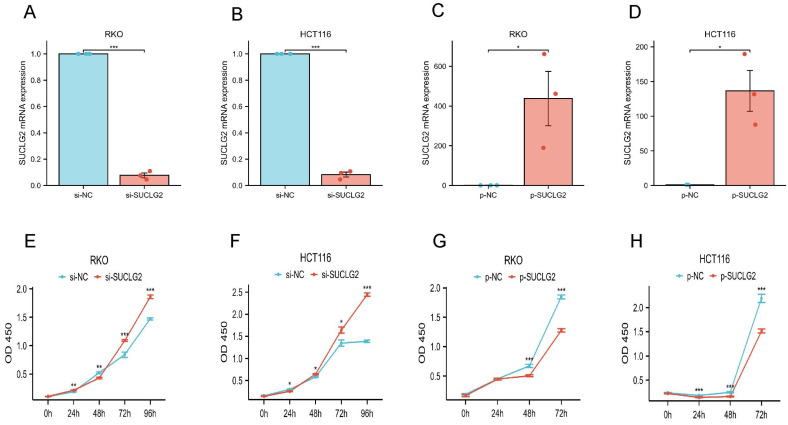
(**A**) The efficiency of siRNAs verified by qRT-PCR in RKO. (**B**) The efficiency of siRNAs verified by qRT-PCR in HCT116. (**C**) The efficiency of overexpression verified by qRT-PCR in RKO. (**D**) The efficiency of overexpression verified by qRT-PCR in HCT116. (**E**) CCK-8 assay of RKO after transfection with si-SUCLG2. (**F**) CCK-8 assay of HCT116 after transfection with si-SUCLG2. (**G**) CCK-8 assay of RKO after transfection with p-SUCLG2. (**H**) CCK-8 assay of HCT116 after transfection with p-SUCLG2. “*” represents *p* < 0.05; “**” represents *p* < 0.01; and “***” represents *p* < 0.001.

To determine its specific role in CRC progression, SUCLG2 was effectively silenced (si-SUCLG2) (Figure 10A,B) or overexpressed (p-SUCLG2) (Figure 10C,D) in the RKO and HCT116 cells, respectively. The results of the CCK-8 assay showed that SUCLG2 silencing promoted the proliferation of RKO and HCT116 cells (Figure 10E,F), whereas SUCLG2 overexpression inhibited their proliferation (Figure 10G,H). Taken together, SUCLG2 inhibited the proliferation of CRC cells in vitro.

## 4. Discussion

Cancer metabolism, which distinguishes cancer cells from normal cells, plays an essential role in various cancer processes. Growing evidence suggests that cancer metabolism is closely linked to carcinogenesis, anticancer immune response, cancer progression, and prognosis [6]. Herein, our pan-cancer analysis indicated that SMOX was highly expressed and SUCLG2 was less expressed in most cancers. These trends were also observed in external data and in vitro experiments involving CRC samples. In addition, SMOX and SUCLG2 showed excellent diagnostic capabilities for CRC. Survival analysis in pan-cancer revealed that SMOX and SUCLG2 acted as independent prognostic factors in multiple cancers, and high SMOX expression or low SUCLG2 expression was associated with worse prognosis. Furthermore, SMOX and SUCLG2 were found to be correlated with TME, TMB, MSI, stemness scores, and drug sensitivity, and involved in various carcinogenic pathways.

Cancer metabolism plays a vital role in fueling rapid growth and is closely associated with cancer prognosis and various biological processes. Bi et al. performed a pan-cancer analysis, focusing on genes involved in glycolysis and oxidative phosphorylation, and found significant differences in patient outcomes based on their metabolic subtypes. In particular, patients with high glycolysis and low oxidative phosphorylation exhibited worse prognosis, and different metabolic subgroups also displayed varying sensitivity to treatment [38]. Deng et al. identified the gene encoding pyruvate dehydrogenase E1 component subunit α to be crucial for reprogramming glucose metabolism in cancer cells; furthermore, high expression level of this gene was reported to be a prognostic risk factor for many cancers [39]. A positive correlation evidently exists between high carbohydrate and energy metabolism and epithelial–mesenchymal transition in various cancers. In addition, cancer metabolism has been linked to prognosis in different TMEs [40].

SMOX and SUCLG2 participate in cancer development and initiation. Studies have indicated that high SMOX expression is an independent prognostic risk factor and that suppressing SMOX expression can inhibit CRC cell proliferation and invasion [41]. SMOX expression has been found to gradually increase in normal liver, chronic hepatitis, and hepatocellular carcinoma, and it is linked to hepatocellular carcinoma prognosis. SMOX also plays a role in regulating the phosphatidylinositol 3′-kinase/protein kinase B signaling pathway in liver cancer cells, affecting cancer cell growth [42]. Chen et al. suggested that SMOX is associated with changes in immune cell infiltration characteristics in TME and prognosis of lung cancer [43]. On the other hand, decreased expression of SUCLG2 can enhance the inhibition of TGF-β signal transduction in cancer stroma through metformin, affecting the promoting effect of TME on cancer cell growth [44]. In this study, SMOX and SUCLG2 were differentially expressed in pan-cancer tissues and associated with cancer prognosis. In in vitro experiments, SMOX and SUCLG2 exhibited the same expression trend in CRC as observed in bioinformatics study. Pathway enrichment analysis involving gene set enrichment analysis revealed that SMOX and SUCLG2 regulate drug metabolism, antigen presentation, and other related pathways, indicating their close relationship with cancer immunity and drug sensitivity. These results are consistent with those of previous studies.

Cancer metabolism is complex and diverse, differing from the metabolic patterns of normal cells. Immune cells, such as activated T cells, regulatory T cells, neutrophils, and other rapidly proliferating immune cells, also undergo metabolic changes [45]. The metabolic competition between cancer cells and immune cells profoundly impacts the immune system. Studies suggest that the glycolytic activity of cancer cells limits glucose consumption by T cells, leading to T cell exhaustion and cancer immune escape [46]. Even when there are sufficient amounts of cancer antigen for T cell recognition, cancers can suppress the function of cancer-infiltrating T cells via competitive glucose uptake [47]. In addition, cancer metabolism-produced lactate reduces pH levels in TME, potentially favoring cancer progression by suppressing cancer immunity [48]. Therefore, there exists a significant relationship between cancer metabolism and cancer immunity. Our findings highlight that SMOX and SUCLG2 are significantly correlated with cancer immunity.

In the context of immunotherapy response prediction, MSI and TMB are commonly used. MSI refers to the failure of the DNA mismatch repair mechanism during DNA replication, leading to alterations in microsatellite length. Patients with high MSI are more responsive to immune checkpoint inhibitors [49]. On the other hand, TMB represents the total number of mutations in a cancer specimen, resulting in the generation of neoantigens on cancer cell surfaces. Higher TMB leads to increased immunogenicity and enhances T cell recognition and cancer cell destruction [50]. Our results indicate an association between SMOX, SUCLG2, MSI, and TMB in various cancers, suggesting the potential of SMOX and SUCLG2 as immunotherapy predictors.

Studies have identified six primary cancer immunophenotypes. C1 is characterized by increased angiogenic gene expression, elevated proliferation rates, and Th2 cells favoring adaptive immune infiltration. C2 exhibits the highest M1/M2 macrophage polarization, superior T cell receptor diversity associated with proliferation rate, and strong CD8 signaling. C3 shows upregulation of Th17 and Th1 genes, low or moderate cancer cell proliferation, reduced aneuploidy, and overall somatic copy number alterations. C4 is characterized by macrophage traits, higher M2 response, and suppressed Th1. C5 exhibits the lowest lymphocyte and the strongest macrophage response, predominantly driven by M2 macrophages. C6 exhibits the highest TGF-β signature, high lymphocyte infiltration, and an even distribution of type I and II T cells [51]. In pan-cancer, we found that SMOX was most highly expressed in C5 and closely associated with resting NK cells, while SUCLG2 was most highly expressed in C3 and associated with T follicular helper cells, resting dendritic cells, activated NK cells, regulatory T cells, resting memory CD4 T cells, and monocytes. These findings were consistent with the identified immunophenotypes. Studies have demonstrated the prognostic significance of NK cell infiltration in cancer tissues across several cancers, with reduced NK cell function associated with poorer outcomes [52]. T follicular helper cells produce interleukin-21 and affect T cell proliferation, viability, cytokine production, and cytotoxic function; moreover, they are crucial for the efficacy of anti-PDL1 therapy [53]. Dendritic cells play a key role in the initiation and regulation of innate and adaptive immune responses. CD70 on dendritic cells promotes CD8+ T cell initiation, differentiation, and anticancer immunity [54]. Regulatory T cells maintain immune homeostasis by suppressing hyperactive immune responses and can induce cancer escape through anergy and immunosuppression during cancer development [55]. Resting CD4 memory cells are associated with overall survival in various cancers, including breast cancer, endometrial cancer, and CRC [56,57,58]. Monocytes bridge the gap between innate and adaptive immune responses and affect TME by inducing immune tolerance and angiogenesis and facilitating cancer cell spread, influencing the efficacy of immunotherapy in patients with cancer [59].

In this study, we further investigated the association of SMOX and SUCLG2 with CRC. Our findings revealed a close relationship between SMOX, SUCLG2, clinical characteristics, cancer immunity in CRC. Furthermore, SMOX and SUCLG2 demonstrated excellent predictive ability for CRC diagnosis and prognosis. Based on our analysis, SMOX and SUCLG2 have good diagnostic value in both CRC tissues and blood. Research confirmed that SMOX promotes colorectal cancer [41]. Our research suggests that SUCLG2 may inhibit CRC. This indicated that SMOX and SUCLG2 play an important role in the diagnosis and development of CRC. SMOX deletion is reportedly linked to worsened colitis, colitis-associated CRC, and intestinal microbiota dysregulation [60]. SMOX has also been identified as a prognostic risk factor for CRC [41]. Anastasiya et al. found that SMOX and other polyamine metabolism genes are closely associated with genes regulating metabolic reprogramming, inflammation, and cell proliferation in CRC [61]. Shian-Ren Lin et al. found that Knockdown of SUCLG2 reduced prostate tumor growth in a xenograft model [17]. In our analysis, SUCLG2 emerged as a prognostic risk factor for PRAD. This further reflects the different roles of SUCLG2 in different cancers. Cho et al. observed a significant association between SUCLG2 and CRC while investigating the relationship between citric acid cycle single nucleotide polymorphisms and CRC [62]. Further studies have provided evidence that tricarboxylic acid cycle-related genes, such as SUCLG2, are closely linked to CRC prognosis [63]. Zhang et al. also found that SUCLG2 expression was downregulated in multiple CRC subtypes and associated with ribosomal biosynthesis in cancer cells as well as CRC prognosis [64]. However, there was few research on the proliferation of SUCLG2 in CRC. Our study found that SUCLG2 has the potential to inhibit CRC.

## 5. Conclusions

To summarize, our pan-cancer analysis of SMOX and SUCLG2 demonstrated their close association with clinical characteristics, prognosis, cancer immunity, cancer cell characteristics, and drug sensitivity in various cancers. We performed additional exploration specifically in CRC tissues to further elucidate the relationship between SMOX, SUCLG2, and CRC. It is notable that our study primarily relied on bioinformatics analysis, with in vitro experiments validating gene expression trends and the potential to inhibit CRC of SUCLG2,. In the future, we aim to further investigate the molecular mechanisms of SMOX and SUCLG2 in cancer and potentially offer new diagnostic and treatment approaches for cancers.

## Data Availability

The data that support the results of current study is available on TCGA, GEO and other databases. The datasets used and/or analyzed during the current study are available from the corresponding author on reasonable request.

## Supplementary Materials

The following supporting information can be downloaded at: https://www.mdpi.com/article/10.3390/cimb47060465/s1.

## Abbreviations

The following abbreviations are used in this manuscript:

ACC	Adrenocortical carcinoma
BLCA	Bladder Urothelial Carcinoma
BRCA	Breast invasive carcinoma
CCK-8	Cell Counting Kit-8
CESC	Cervical squamous cell carcinoma and endocervical adenocarcinoma
CHOL	Cholangiocarcinoma
COAD	Colon adenocarcinoma
CRC	Colorectal cancer
DLBC	Lymphoid Neoplasm Diffuse Large B-cell Lymphoma
ESCA	Esophageal carcinoma
GBM	Glioblastoma multiforme
GEO	Gene Expression Omnibus
HNSC	Head and Neck squamous cell carcinoma
KICH	Kidney Chromophobe
KIRC	Kidney renal clear cell carcinoma
KIRP	Kidney renal papillary cell carcinoma
LAML	Acute Myeloid Leukemia
LGG	Brain Lower Grade Glioma
LIHC	Liver hepatocellular carcinoma
LUAD	Lung adenocarcinoma
LUSC	Lung squamous cell carcinoma
MESO	Mesothelioma
MSI	Microsatellite instability
OV	Ovarian serous cystadenocarcinoma
PAAD	Pancreatic adenocarcinoma
PCPG	Pheochromocytoma and Paraganglioma
PRAD	Prostate adenocarcinoma
READ	Rectum adenocarcinoma
SARC	Sarcoma
SKCM	Skin Cutaneous Melanoma
STAD	Stomach adenocarcinoma
TCGA	The Cancer Genome Atlas
TGCT	Testicular Germ Cell Tumors
THCA	Thyroid carcinoma
THYM	Thymoma
TMB	Tumor mutational burden
UCEC	Uterine Corpus Endometrial Carcinoma
UCS	Uterine Carcinosarcoma
UCSC	University of California Santa Cruz
UVM	Uveal Melanoma
