# Supplementary material for: Metabolism-Related Genes SMOX and SUCLG2 as Immunological and Prognostic Biomarkers in Colorectal Cancer: A Pan-Cancer Analysis
Source: Curr Issues Mol Biol. 2025 Jun 17;47(6):465. doi: 10.3390/cimb47060465 (PMC12191581; doi:10.3390/cimb47060465)
Supplement: Supplementary file 1 [file cimb-47-00465-s001.zip › cimb-3638865-supplementary.pdf]

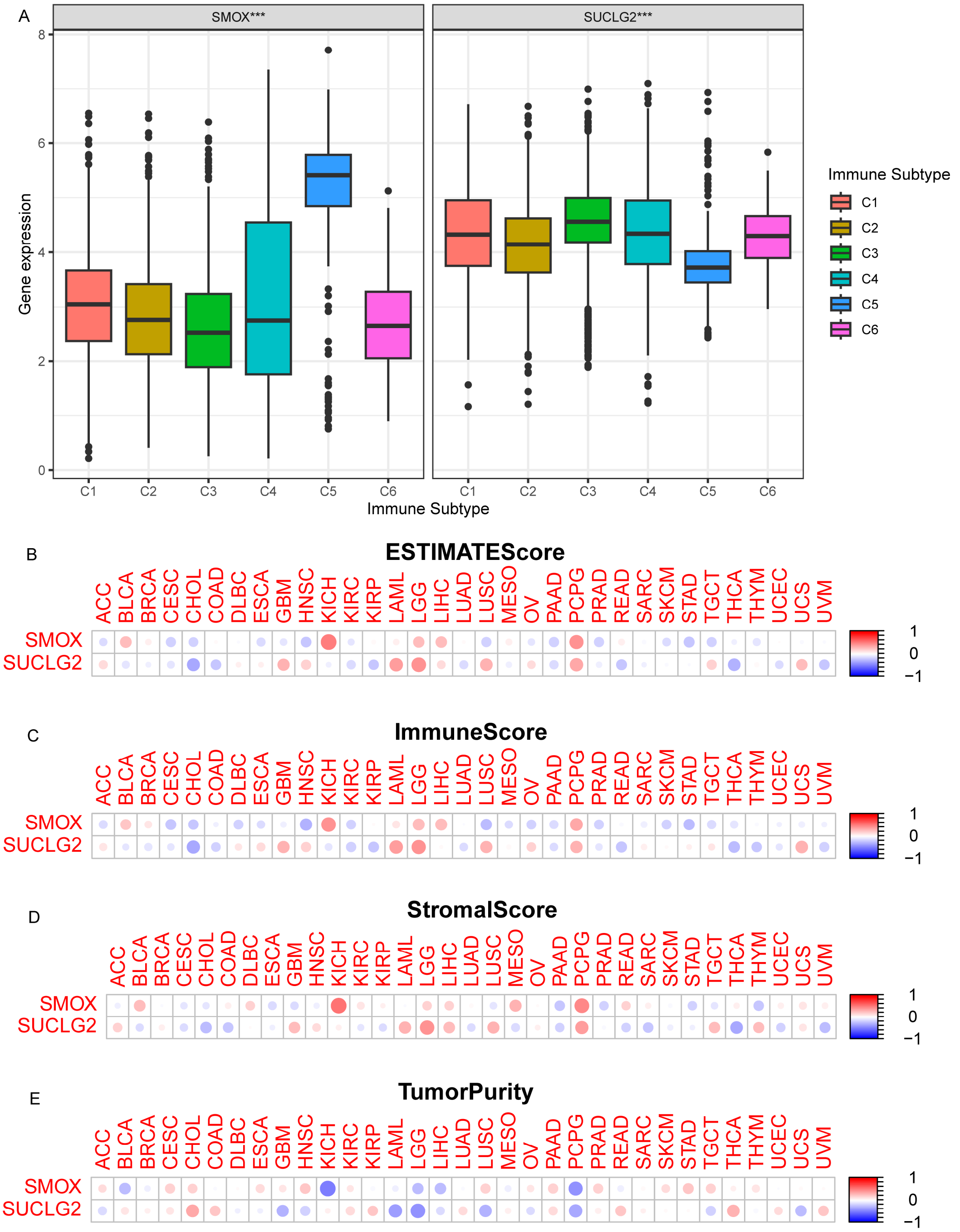

Figure S1. (A) SMOX and SUCLG2 expression correlation with six phenotype - Immune subtypes from UCSC. (B − E) Correlation of SMOX and SUCLG2 with ESTIMATE, immune, and stromal scores and with tumor purity.

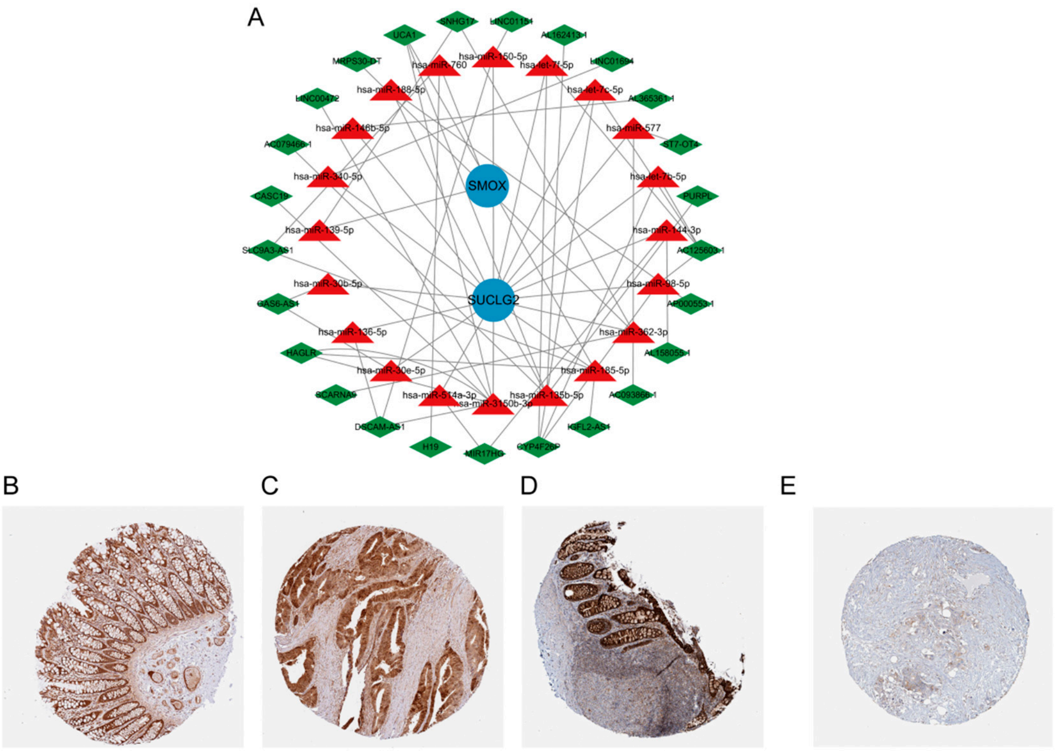

Figure S2. (A) ceRNA network of SMOX and SUCLG2 in CRC. SMOX expression in (B) normal colorectum and (C) CRC. SUCLG2 expression in (D) normal colorectum and (E) CRC.
